# Neddylation Inhibition Causes Impaired Mouse Embryo Quality and Blastocyst Hatching Failure Through Elevated Oxidative Stress and Reduced IL-1β

**DOI:** 10.3389/fimmu.2022.925702

**Published:** 2022-07-04

**Authors:** Guangping Yang, Jianhua Chen, Yanni He, Hui Luo, Hongxia Yuan, Liangliang Chen, Lingli Huang, Fei Mao, Saifei Hu, Yun Qian, Congxiu Miao, Ruizhi Feng

**Affiliations:** ^1^State Key Laboratory of Reproductive Medicine, Nanjing Medical University, Nanjing, China; ^2^Reproductive Medical Center of the Second Affiliated Hospital of Nanjing Medical University, Nanjing, China; ^3^Department of Reproductive Genetics, Heping Hospital of Changzhi Medical College, Institute of Reproduction and Genetics of Changzhi Medical College, The Reproduction Engineer Key Laboratory of Shanxi Health Committee, Changzhi, China

**Keywords:** blastocyst hatching, inflammatory factor, Neddylation, MLN4924 (pevonedistat), oxidative stress

## Abstract

Mammalian blastocyst hatching is an essential prerequisite for successful embryo implantation. As the rate-limiting step of current assisted reproductive technology, understanding the key factors regulating blastocyst hatching would be significantly helpful to improve the performance of the assisted reproductive practice. In early embryo development, the fine-tuned elimination of maternal materials and the balanced protein turnover are inevitable for the competent to hatch and implant into endometrium. Neddylation, a ubiquitination-like protein modification, has been shown to be involved in oocyte maturation and early embryo development. In this study, aiming to discover an unknown role of neddylation in the blastocyst hatching process, we provided functional evidence of neddylation in mammalian embryo quality and blastocyst hatching. Treatment with MLN4924, a specific neddylation inhibitor, lowered the embryo quality and dramatically reduced the hatching rate in mouse blastocysts. The transcriptional profile showed the upregulation of oxidative stress-related genes and aberrant expression of immune-related genes. The elevated oxidative stress was validated by qPCR and markers of apoptosis, DNA damage, reactive oxygen species, and cytoskeleton. Moreover, we found the secreted IL-1β level was reduced in an NF-κB-independent manner, leading to the final poor embryo quality and blastocyst hatching failure. This is the first report of neddylation being of great importance in the mammalian blastocyst hatching process. Further investigations uncovering more detailed molecular mechanisms of neddylation regulation in blastocyst hatching would greatly promote not only the understanding of this crucial biological process but also the clinical application in reproductive centers.

## Introduction

In the efforts of mankind to defeat diseases, sophisticated technologies were invented and glorious achievements have emerged. Represented by IVF-ET (*In vitro* fertilization-embryo transfer), assisted reproductive technologies (ART) fulfill the dreams of numerous infertility patients to become parents. The final event of a successful IVF-ET is embryo implantation into the uterine endometrium. Blastocyst hatching is a necessary early development process prior to embryo implantation and is thought to be the rate-limiting step of current ART practice (1). With the embryo growth, cell mass cells break through the zona pellucida (ZP), which is no longer needed as a protective shell, and enables the cell fusion with endometrium to make successful embryo implantation. In humans, blastocyst hatching takes place on day 6 to day 7 after fertilization, and in mice it occurs on day 5 to day 6. ZP is the main obstacle for cells to overcome, thus secretory zonalytic proteases are produced by the embryo and the uterine cavity. Blastocyst hatching failure may occur when the physicochemical property of ZP changes. Data have shown that transfer of hatched embryos, rather than expanded but not hatching embryos, have better pregnancy outcomes (1). Assisted hatching techniques are applied to improve the embryo competence and implantation success rate. Conventionally, ZP are partially thinned by mechanical/chemical/biological digestion, laser cutting, or piezo-mediated slotting (2). However, there are unfavorable side effects such as embryo impairment and the increasing tendency of monozygotic twins (3, 4).

Due to the essential role of blastocyst hatching in early embryo development and implantation, its regulation has been intensively investigated for decades. Factors influencing blastocyst hatching have been shown to involve hydrostatics in blastocyst cavity, cytokines, growth factors, and hormones. Previous studies have shown that by modulating proteases, pro-inflammatory and anti-inflammatory cytokines improved hatching rates (5), which may reflect the *in vivo* interaction between the blastocyst and the microenvironment/maternal immune system. It was published in 2021 that IL-1β (interleukin-1β) was indispensable for mouse blastocyst hatching by regulating the hatching-associated protease ISP2 (6). The same group also reported that supplementation of IL-1β to 8-cell embryos greatly increased their hatching rate, together with the enhanced expressions and activities of zonalytic proteases, cathepsin-L and cathepsin-B, which were colocalized with IL-1β in trophectodermal projections of the golden hamster hatching blastocysts (7). However more detailed molecular mechanisms remain poorly understood.

Maternal material storage and fine-tuned degradation are unique characteristics and pivotal biological processes in early embryo development and the prerequisite of successful implantation. Post-translational modification is a novel regulation mode in oocyte maturation and embryo development, catching more and more attention by researchers in recent years (8). As the main pathway of protein degradation, ubiquitination and its new forms have been well studied. Neddylation, a ubiquitination-like modification, has been shown to participate in a large variety of cellular processes and to be involved in modulating cell-cell and cell-microenvironment interactions (9, 10), for example, immune-related conditions including inflammation and cancer (11). Previous works also showed that neddylation plays a significant role in mammalian oocyte maturation and early embryonic development (12, 13). Blastocyst hatching is the essential step but the exact function of neddylation has not been studied yet.

In this study, aiming to explore the role of neddylation in blastocyst hatching, we used the specific neddylation inhibitor MLN4924 (pevonedistat) in mouse blastocysts to investigate the potential role of neddylation in mammalian blastocyst hatching. We also performed the transcriptional profile to analyze the downstream change and the underlying mechanisms. Key molecules were detected by immunofluorescence regarding a series of biological processes in blastocysts. Our results showed that neddylation plays important roles in mouse embryo quality and blastocyst hatching and provides a novel angle for further study of the etiology of implantation failure.

## Results

### MLN4924 Treatment in Mouse Blastocysts Led to Growth Restriction, Developmental Abnormality, and Blastocyst Hatching Failure

**Figure 1:**
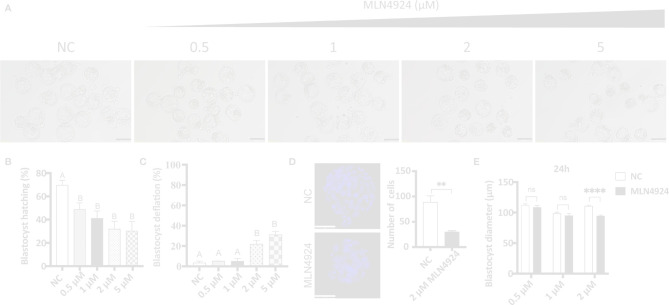
Abnormal embryo growth, development, and impaired blastocyst hatching after MLN4924 treatment in mouse embryos. **(A)** Images of blastocysts in the control group and MLN4924-treated groups. Blastocysts were cultured in a medium with different concentrations of MLN4924 (0.5 μM, 1 μM, 2μM, and 5μM) or without MLN4924. The number of blastocysts in each group is 90. Scale bar for 200 μm. **(B)** Blastocyst hatching rate of control and MLN4924-treated embryos. Error bars represent the standard error. **(C)** Shrinkage blastocyst rate of control (n = 60) and MLN4924-treated embryos (n = 60). Error bars represent the standard error. **(D)** Images of blastocysts nucleus in the control group and MLN4924-treated group (2 μM). The histogram is the cell count of the control group and the MLN4924-treated blastocysts. Scale bar for 50 μm. **(E)** Diameters of blastocysts in NC and MLN4924-treated groups after 24 h.

Mouse embryos were acquired by IVF and cultured *in vitro* until they reached the blastocyst stage. Then gradient supplements (0 μM, 0.5 μM, 1 μM, 2 μM, and 5 μM) of MLN4924 were given for 24 h. The morphologies of blastocysts are shown in **Figure 1A**. We found blastocyst hatching rates were decreased dramatically in MLN4924-treated groups. In NC (negative control, 0 μM) group, the hatching rate were 69.72% ± 7.09%. In MLN4924-treated groups, the hatching rates were 48.78% ± 9.72% in 0.5 μM, 41.13% ± 10.97% in 1 μM, 31.94% ± 11.50% in 2 μM, and 30.28% ± 14.00% in 5 μM, respectively (**Figure 1B**). Each of the gradient concentrations reached a statistically significant different level compared to the NC group (NC vs. 0.5 μM, P = 0.0394, NC vs. 1 μM, P = 0.0193, NC vs. 2 μM, P = 0.0084, NC vs. 5 μM, P = 0.0121). Embryo growth was restricted in a dose-dependent manner. The fraction of deflated blastocyst increased from 3.33% ± 2.89% in the NC group to 5.09% ± 0.15% in 0.5 μM, 5.00% ± 5.00% in 1 μM, 21.97% ± 6.06% in 2 μM, and 31.11% ± 5.36% in 5 μM, as shown in **Figure 1C**. We counted cell numbers as the marker of embryo quality in both NC and MLN4924-treated blastocysts. A significant reduction was observed from 88.60 ± 28.43 in the NC group to 30.00 ± 4.796 in the MLN4924-treated group, P=0.0019, shown in **Figure 1D**. From these data we thought the 5 μM was a saturated concentration. We then measured the diameters of blastocysts in all groups except the 5 μM concentration. As shown in **Figure 1E**, for 24 h treatment, only the 2 μM group showed a significant decrease in blastocyst diameter compared to the NC group, from 110.84 μm ± 9.34 μm to 94.34 μm ± 11.83 μm, P < 0.0001 (112.00 μm ± 11.84 μm in the NC group and 108.50 μm ± 11.17 μm in 0.5μM in the MLN4924 group, P=0.0953; 98.64 μm ± 12.30 μm in the NC group and 94.92 μm ± 12.54 μm in 1 μM in the MLN4924 group, P=0.1060). For 48 h treatment, shown in **Figure S1**, all three treatment groups reached the significant level of P < 0.0001. In 0.5 μM, the diameters decreased from 129.4 μm ± 20.35 μm to 114.6 μm ± 17.11 μm. In 1 μM, the diameters decreased from 125.9 μm ± 18.90 μm to 104.7 μm ± 17.67 μm. In 2 μM, the diameters decreased from 126.4 μm ± 22.60 μm to 88.90 μm ± 11.84 μm.

As a result, MLN4924 treatment in mouse blastocysts caused growth restriction, lowered embryo quality, and hatching impairment. Considering the decreasing content, variation degree, and morphological changes, we chose 2 μM MLN4924 as the representative treatment group for subsequent experiments.

### Transcriptome Profile Analysis in NC and MLN4924-Treated Blastocysts

**Figure 2:**
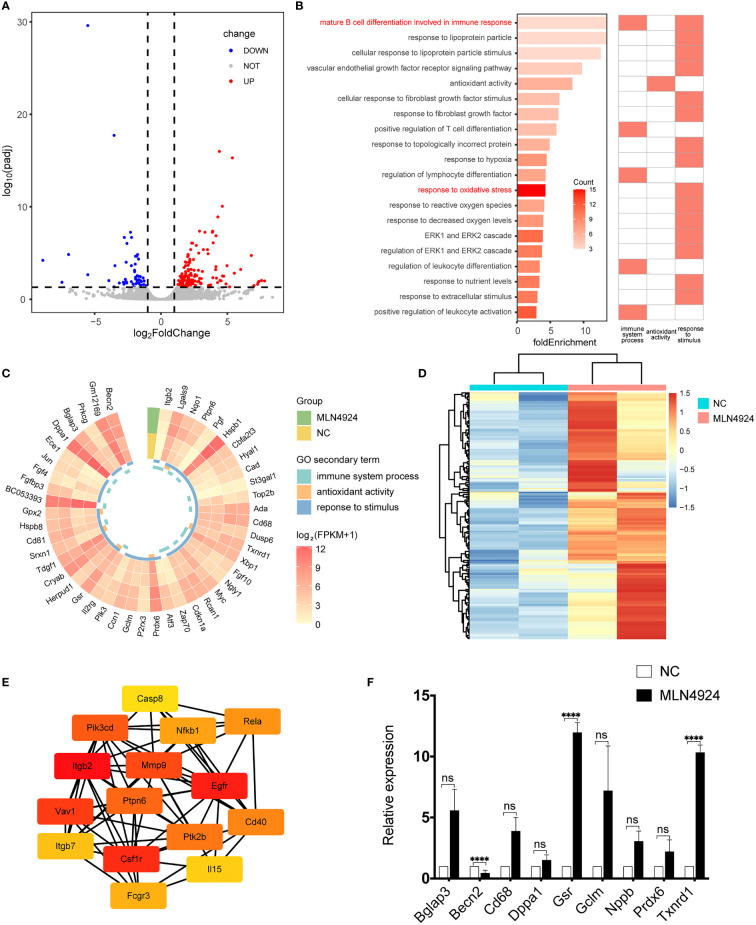
Transcriptional profile analysis and qPCR validation of NC and MLN4924-treated blastocysts. **(A)** The volcano plot showed the differentially expressed genes (DEGs) between the MLN4924-treated group and the control group. Each dot represents one gene. **(B)** GO enrichment analysis of DEGs, all terms that are related to the immune system process, antioxidant activity, and response to stimulus. **(C)** Gene expression level in related terms. **(D)** Heatmap of genes in terms associated with immunity, cytokines, and stimulus from GSEA enrichment analysis. **(E)** Hub genes identified from the PPI network from immune, cytokines and stimulus-related genes by cytoHubba plug-in. **(F)** Validation of DEGs by qRT-PCR.

Single-cell transcriptome profile was performed in the 2μM MLN4924-treated group and the NC group. Correlation analysis and principal component analysis (**Figures S2A, B**) were used to evaluate the sample quality and then exclude the abnormal sample MLN3 for further analysis. There were 221 DEGs, of which 62 were downregulated and 159 were upregulated, in the blastocysts of the MLN4924-treated group as determined using the DESeq2 package with a threshold of adjusted P-value <0.05 and |log_2_FoldChange|>1 (**Figure 2A**).

GO analysis was carried out on these DEGs and the WEGO database was used to cluster GO annotations. We found some significant items of biological process, cellular component, and molecular function enriched in the secondary annotations, such as immune system process, antioxidant activity, and response to stimulus (WEGO id: WEGOID49935334150852). We selected specific categories involved in these items and their fold enrichment score was calculated. We found that the top one was material B cell differentiation involved in immune response, relating to both immunity and stimulus (**Figure 2B**) and response to oxidative stress reached the maximum number of genes, which hints at the possible mechanism of neddylation in mammalian blastocyst hatching. Whereupon we investigate the expression pattern of genes involved in all 20 categories (n = 47), 68% of them (n = 32) were upregulated in the MLN4924-treated group Of these upregulated genes, 82.1% (n = 23) had an average FPKM greater than 10 (**Figure 2C**), which was considered to be highly expressed.

To support the results of GO analysis, GSEA analysis was performed using all 13,078 expressed genes, and 196 genes in seven enrichment terms (**Figure S1C**) related to immunity, cytokines, and stimulus were selected for the heatmap. The expression of these genes demonstrated an upregulation trend in the MLN4924 treatment group (**Figure 2D**) and Itgb2, Cd81, Hyal1, Gpx2, Cd68, Ada, Lgals9, Pgf, Hspb1, and Ptpn6 were found in GO and GSEA results. Protein-protein interaction was performed and hub genes were identified by CytoHubba, including p65 (also known as Rela) and NF-κB (**Figure 2E**). The differentially expressed genes were validated by qPCR, as shown in **Figure 2F**, indicating an overall elevation of oxidative stress.

### Neddylation Inhibition Caused Elevated Oxidative Stress in MLN4924-Treated Blastocysts

**Figure 3:**
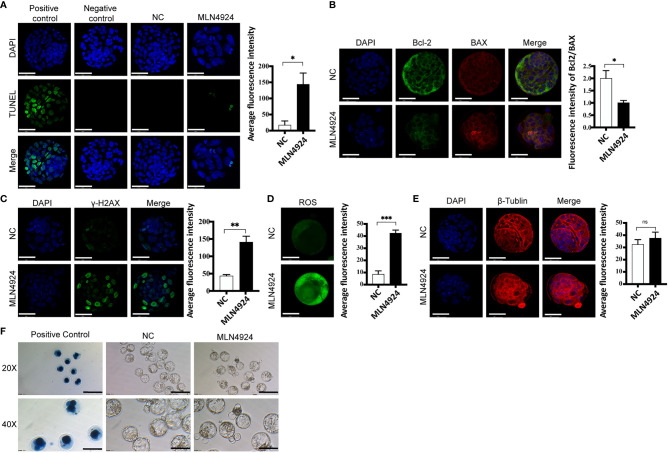
Neddylation block elevated oxidative stress in blastocysts. **(A)** TUNEL assay detecting late cell apoptosis in NC (n = 30) and MLN4924-treated blastocysts (n = 30). Positive control: blastocyst treated with DNase I Negative control: blastocyst without TdT enzyme. The signal intensities were increased in MLN4924-treated blastocysts comparing with NC. Scale bar for immunofluorescence image is 50 μm. **(B)** Immunofluorescence of Bcl-2 and BAX (Bcl2 Associated X Protein) in NC (n = 30) and 2 μM MLN4924-treated blastocysts (n = 30). Green stained blastocysts represent Bcl-2, red-stained blastocysts represent Bax. The histogram is the fluorescence intensity ratio of Bcl 2 and BAX, the ratio of the control group was higher than that of the MLN4924-treated blastocysts. **(C)** Immunofluorescence was used to capture representative γH2AX focus images from NC (n = 30) and MLN4924-treated blastocysts (n = 30). Increasing fluorescence intensity was detected in the MLN4924. **(D)** ROS (Reactive Oxygen Species) assay evaluating mitochondrial function in NC (n = 30) and MLN4924-treated group (n = 30). The cell localization changed and fluorescence signal intensities were increased in MLN4924-treated blastocysts compared with NC. **(E)** Intensity of β-tubulin (red) immunofluorescence reflecting cytoskeleton function. The fluorescence signal intensities showed no significant difference in MLN4924-treated blastocysts (n = 30) compared with NC (n = 30). **(F)** Cell viability tested with Trypan blue staining. Dead cells were stained with blue, showing in PC. Both NC (n = 60) and MLN4924-treated blastocysts (n = 60) were viable. Scale bar for bright filed image is 200μm.

From the transcriptome profiles and enrichment analysis, we then tested the overall oxidative stress in mouse blastocysts with several markers from different aspects. We tested the apoptotic status of the blastocysts with TUNEL assay. Immunofluorescence showed apoptotic signals in a few cells in MLN4924-treated blastocysts, whereas the NC group had almost no apoptosis at all (**Figure 3A**). More apoptosis markers were detected and we found the ratio of Bcl2/BAX was decreased from 2.003 ± 0.54 in the NC group to 1.00 ± 0.16 in the MLN4924-treated group, as shown in **Figure 3B**. To evaluate the extent of DNA damage, we detected the level of γ-H2AX, a DNA double-strand break marker with immunofluorescence. A significant increase of γ-H2AX was found in the MLN4924-treated group compared with the NC group, and the signal intensities were 141.00 ± 29.45 in the MLN4924-treated group and 43.29 ± 6.95 in the NC group, P=0.0050 (**Figure 3C**). The embryonic reactive oxygen species (ROS) level reflects the overall oxidative stress and mitochondrial function. We found a significant increase in ROS signal intensity in the MLN4924-treated group (42.41 ± 4.40) compared with the NC group (8.59 ± 4.76), P=0.0008 (**Figure 3D**). To evaluate cytoskeleton status, we detected β-tubulin and found a slack microtubule network in the MLN4924-treated blastocyst group (**Figure 3E**). In addition, we performed Trypan blue staining to evaluate cell viability and potential cytotoxicity of the MLN4924 treatment group. No blue stain was found in MLN4924-treated blastocysts, indicating that there were no dead cells as a result of neddylation block (**Figure 3F**, n = 30).

Thus, the oxidative stress of blastocysts was increased in the MLN4924-treated group, indicating that biological function disturbance existed, which may result in blastocyst hatching failure.

### Altered IL-1β Secretion in MLN4924-Treated Blastocysts Regulated in an NF-κB-Independent Manner

**Figure 4:**
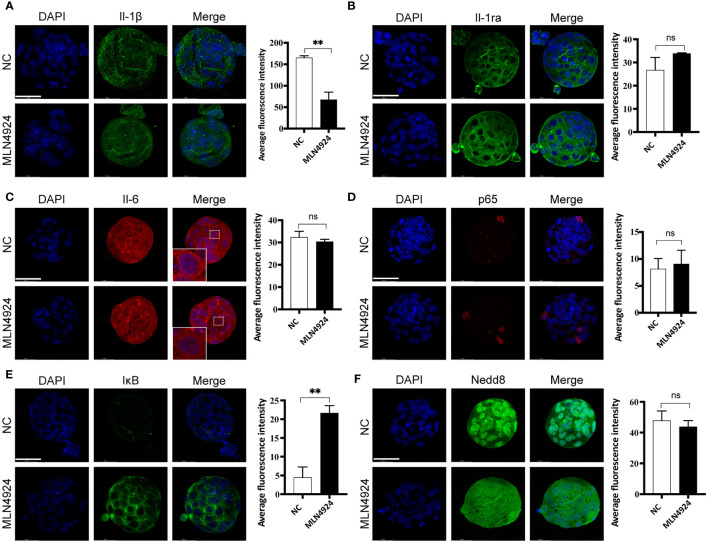
Secreted IL-1β reduced in an NF-κB-independent manner after neddylation inhibition. **(A)** Immunofluorescence of IL-1β (green) in NC (n = 30) and the MLN4924-treated blastocysts (n = 30). DPAI-labeled DNA is shown in blue. The fluorescence signal intensities were decreased in MLN4924-treated blastocysts compared with NC. **(B)** Representative images of IL-1ra (green) from NC (n = 30) and the MLN4924-treated groups (n = 30). No significant difference in fluorescence intensity between the two groups. **(C)** Immunofluorescence of IL-6 (red) in NC (n = 30) and the MLN4924-treated groups (n = 30). The intensity of the two groups showed no significant difference. **(D)** Immunofluorescence of p65 (red) in NC (n = 30) and the MLN4924-treated blastocysts (n = 30). The fluorescence signal intensities showed no significant difference in MLN4924-treated blastocysts compared with NC. **(E)** Immunofluorescence of IκB (green), a member of the NF-κB signal pathway, in NC (n = 30) and the MLN4924-treated blastocysts (n = 30), showing significant accumulation in the MLN4924-treated group. **(F)** Immunofluorescence of Nedd8 (green) in NC (n = 30) and the MLN4924-treated blastocysts (n = 30). Compared with the nuclear localization of Nedd8 in NC group, blastocysts treated with MLN4924 lost the original cellular localization and diffused into the cytosol.

Inflammatory cytokines were reported to regulate blastocyst hatching, which was in accordance with our transcriptional analysis. We detected expression of IL-1β, its natural antagonist IL-1ra and IL-6 in NC and MLN4924-treated blastocysts. Immunofluorescence showed the signal dots of IL-1β with intercellular locations and the intensity of IL-1β dots were reduced in the MLN4924-treated group (165.2 ± 7.88) compared with NC (67.47 ± 30.57), P=0.0058 (**Figure 4A**). Intracellular expression of IL-1ra showed no significant difference between the two groups, although a slightly more condensed green staining was observed (26.72 ± 9.42 in the NC group and 33.85 ± 0.53 in the MLN4924-treated group, P=0.2608, **Figure 4B**). The fluorescence intensity of IL-6 was not obviously changed (32.31 ± 4.69 in the NC group and 30.38 ± 1.81 in the MLN4924-treated group, P=0.5421) but its cellular localization was altered. In the NC group, the IL-6 signal enriched toward the cell membrane boundary while after MLN4924 treatment, the signal was diffused into the cytoplasm (**Figure 4C**). The NF-κB transcription factors have been illustrated to play a pivotal role in inflammatory response and cytokine/chemokine production (14). Additionally, as shown in **Figure 2E**, using protein-protein interactions, we found Rela (also known as p65) and Nfkb1 (also known as Nfkb) were members of the top 15 hub genes. A previous study has shown that MLN4924 treatment in a neutrophilia mouse model markedly reduced the expression of IL-1β and IL-6 by blocking the activation NF-κB signaling pathway (15). To test if this is the case in our current study, we detected two members of NF-κB signaling pathway, p65 and IκB, in blastocysts of the two groups. To our surprise, in both groups only a few cells in the blastocysts were p65-positive (8.15 ± 3.35 in the NC group and 9.06 ± 4.43 in the MLN4924-treated group, P = 0.7896) and there was no p65 nuclear translocation found, which is the hallmark of activated NF-κB signaling pathway (**Figure 4D**). Although remarkably, accumulation of IκB was found in the MLN4924-treated blastocysts (4.50 ± 4.78 in the NC group and 21.64 ± 3.43 in the MLN4924-treated group, P = 0.0072), meaning that although the inhibitory effect to NF-κB signaling pathway existed, the downstream of NF-κB signaling pathway was not activated in either group (**Figure 4E**). Thus, the reduced IL-1β was probably regulated in an NF-κB-independent manner. Finally, we detected the expression of Nedd8, the “label” of neddylation in both these groups. Distinct cellular localization pattern alteration was found after MLN4924 treatment, although the signal intensities were not changed significantly (47.80 ± 10.84 in the NC group and 43.82 ± 6.96 in the MLN4924-treated group, P = 0.6203, **Figure 4F**), indicating significant changes may take place in Nedd8-related signal pathways.

**Figure 5:**
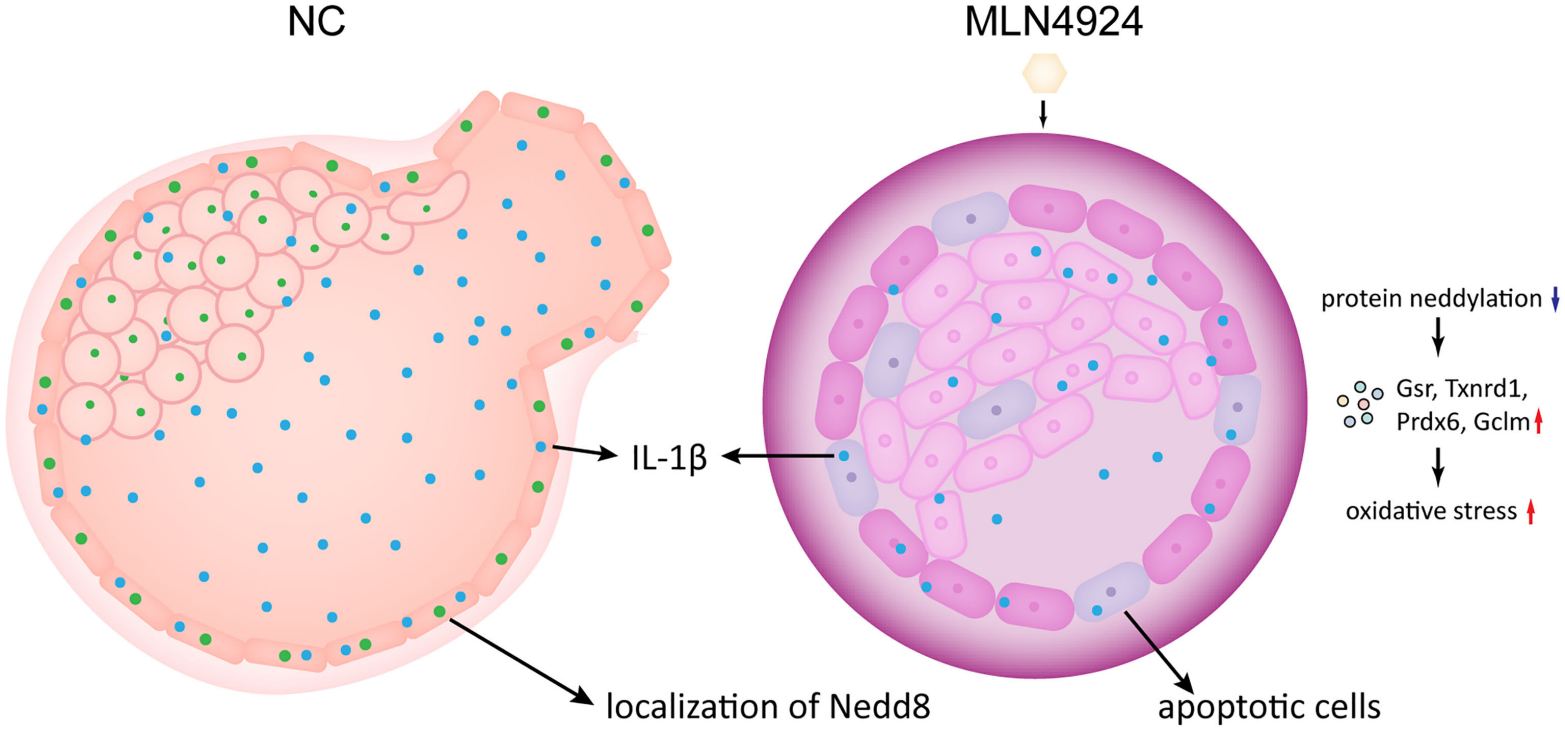
A schematic representation of the neddylation block causing blastocyst hatching failure. With MLN4924 in mouse blastocyst inhibits protein neddylation, leading to putative aberrant protein degradation and turnover, and raise the integral oxidative stress level. At the same time, the overall oxidative stress level increased and the secreted IL-1β reduced, accompanied by elevated DNA damaging, mitochondrial dysfunction and apoptosis. The restricted embryo growth also caused abnormal hydrostatic pressure in the blastocyst. Altogether the final blastocyst hatching failure takes place.

Taken together, our results demonstrated the neddylation inhibition caused by MLN4924 treatment in mouse blastocysts led to a series of phenotypes indicating abnormal growth, development, and the final crucial step, blastocyst hatching. Intensified oxidative stress was identified by transcriptome profile and validated with several markers. Apoptotic cells were found in the MLN4924-treated blastocysts, with DNA damage and dysfunction in biological processes, putatively detrimental effects took place contributing to the insufficient hydrostatic pressure in the blastocyst cavity. The secreted proinflammatory cytokine IL-1β was reduced in the MLN4924-treated blastocysts in an NF-κB-independent manner and caused the impairment of embryo quality and blastocyst hatching competence. These findings are summarized in **Figure 5**.

## Discussions

In this study, we provided evidence that neddylation plays important roles in mouse embryo growth, development and blastocyst hatching. Transcriptional profile showed the upregulation of oxidative stress related genes and aberrant expression of immune-related genes in MLN4924-treated blastocysts, suggesting that altered cytokine expression after neddylation block may increase the stress level leading to the impeded blastocyst hatching progress. Elevated oxidative stress was validated with markers of DNA damaging, cell apoptosis, ROS and cytoskeleton architecture. We also detected the decreased signals of secreted intracellular pro-inflammatory cytokine IL-1β, slightly elevated cytoplasm signal of its natural antagonist IL-1ra and altered expression pattern of IL-6, all of which were regulated after neddylation inhibition in an NF-κB-independent manner. The nuclear localization of Nedd8 disappeared after MLN4924 treatment, indicating that downstream molecules and signal pathways were responsible for blastocyst hatching failure.

Our transcriptional profile analysis showed that in neddylation-inhibited blastocysts, there are upregulated genes involving immune system process, antioxidant activity and response to stimulus. Oxidative stress often leads to detrimental effects regarding DNA, proteins, lipids so that normal biological processes may be disturbed (16). In embryo, the natural low oxygen environment may induce the increase of HIF (hypoxia-inducible factor) to reduce the oxidative stress level for normal early development (17). The validated significantly changed genes and not significant but with clear changing tendency genes indicated that related pathways were activated generally on the whole level of the blastocyst. Oxidoreductases Txnrd1 and Gsr were drastically increased in the MLN4924-treated blastocysts, demonstrating the elevated oxidative stress level after neddylation inhibition. Previous studies have shown that the Txnrd1, Thioredoxin reductase 1, is indispensable for embryogenesis (18). It is noted that a batch of antioxidant activity-related genes showed increasing tendency but did not reach a statistically significant level: Prdx6, Nppb, Gclm, Dppa1, Cd68 and Bglap3, reflecting the overall stress status in the embryo as a result of interfered protein degradation and turnover caused by neddylation inhibition.

Blastocyst hatching is a complex biological process regulated by numerous molecules including proteases, transcription factors, growth factors, and cytokines (5). Different kinds of inflammatory cytokines express in peri-hatching blastocyst and the potential implantation competence were influenced by the complicated regulation of pro-inflammatory and anti-inflammatory factors through modulating proteases as one possible mechanism. Direct functional experimental evidence was reported by knockout studies or *in vitro* studies, showing the significant roles of cytokines in embryo development, blastocyst hatching, and implantation. The decreased level of secreted IL-1β in hatching-impaired MLN4924-treated blastocysts is in accordance with previous published studies. Researchers showed the supplement of pro-inflammatory cytokine IL-1β improved the blastocyst hatching in both mouse and golden hamster embryos by promoting the expressions of related proteases (6, 7). We noticed that the immunofluorescence staining pattern of IL-1β in our study, the sporadic intercellular dots, was different from the previous study (6) and it could be due to the following two reasons. In this previous study, embryos were developed *in vivo* until they reached the morulae stage and freshly recovered by flushing the mouse uterine horns. We acquired our mouse embryo samples by *in vitro* IVF and embryo culture until the blastocyst stage. During the *in vivo* development, embryos receive nutrients and signals from the maternal body and have interactions with the maternal immune system. The signals from the maternal immune system would play as stimuli to make the embryos activate to produce related cytokines. Thus, it is reasonable to have a higher expression and distinct cellular localization of the pro-inflammatory cytokine IL-1β embryos developed *in vivo* than those cultured from IVF procedures *in vitro*. The other reason is from the detecting of IL-1β with antibodies. The IL-1β molecule has two co-existing formats, the 31KD precursor and 17KD mature molecule. Different antibodies in different assays may have a preference to different molecules or both of them. Altogether *in vivo* and *in vitro* cultures may induce different expression intensity and format of IL-1β, so detecting with different antibodies may give distinct experiment results.

Though *in vivo* embryo culture would be a more natural model, the reason we chose to use the IVF culture method is that we wanted to mimic the clinical practice in reproductive centers of treating infertile patients with the IVF-ET assisted reproductive technology. Moreover, previous studies in humans showed that IL-1β is associated with pregnancy outcomes. As early as in 1998, it was reported that the implantation rate could be higher in IVF-ET patients with higher IL-1β level in serum than those with no detectable IL-1β (19). A similar conclusion was found in 2010 showing that serum IL-1β was a predictor of successful implantation and pregnancy in COS controlled ovarian stimulation (COS)/intracytoplasmic sperm injection (ICSI) patients (20). In 2013, researchers found that the IL-1β concentration in follicular fluid of IVF patients with >90% fertilization rate was significantly higher than those with no more than 90% fertilization rate (21). The predictive value of IL-1β concentration was later confirmed by more recent research (22, 23). Apart from the maternal IL-1β, the amount of secreted IL-1β by the embryo in culture media was correlated with human blastomere numbers, reporting in 2012 (24). It was also reported in 2015 that the IL-1β concentration in day 3 culture-conditioned medium was significantly higher in women who achieved pregnancy than those who did not in a total of 27 patients (P<0.001) (25). Similar results were found with more details in mouse experiments, showing that the supplement of IL-1β would improve the embryo quality by generating more cells in the blastocysts (26). Our current study corroborated these previous findings and provided further knowledge of IL-1β and its important roles in mammalian embryo development and blastocyst hatching.

Furthermore, we investigated the underlying molecular mechanism of the elevated oxidative stress and reduced IL-1β. It is generally recognized that the production of IL-1β was regulated by the NF-κB signaling pathway as an inflammatory response (14). A previous study has shown that the NF-κB signaling pathway was activated during the maternal-zygote transfer at the two-cell stage in mice. Distinct p65 nuclear translocation was reported in two-cell and four-cell stages and inhibition of the NF-κB signaling pathway led to embryo arrest at the two-cell stage (27, 28). The function of NF-κB signaling pathway in mammalian blastocysts was rarely studied. The only publication we found was performed in the golden hamster. By treating blastocysts with NF-κB inhibitors, researchers found that NF-κB signaling system was required for blastocyst hatching (29). However, in our study, we found the NF-κB signaling pathway was silenced by no nuclear translocation of p65. This could be due to the species differences between mice and golden hamsters and further studies are needed for confirmation. Moreover, our results of cell counting were in accordance with the previous study of the effect of IL-1β to blastocyst cell numbers and embryo quality (26). Previous studies have shown that induction of autophagy would promote the blastocyst development (30, 31). In the above mentioned 2020 study, the authors found that in the VGX-1027 (IL-1β inhibitor) treated group, autophagy‐related factors Atg5 and LC3 were dramatically reduced, indicating that these factors were the downstream targets regulated by IL-1β (26). We speculated that the autophagy status in the MLN4924-treated blastocysts was inhibited, thus leading to a deteriorated embryo quality. More exploration will be performed in our further study to test our speculation and discover more detailed regulation mechanisms. Our study is the first report about the participation of neddylation in mammalian blastocyst hatching. Neddylation could be a novel regulation pathway of blastocyst development potential and may be involved in some challenges the embryo meets in ART practice such as cryodamage in vitrification or other disturbance of microenvironment both *in vitro* and *in vivo* (32).

In summary, we propose the hypothesis that neddylation inhibition causes the interference of protein degradation and turnover, after which oxidative stress levels increase in the blastocyst. Dysfunction in biological processes led to putatively detrimental effects contributing to the insufficient hydrostatic pressure in the blastocyst cavity. The secreted proinflammatory cytokine IL-1β was reduced in the MLN4924-treated blastocysts in an NF-κB-independent manner and caused the impairment of embryo quality and finally blastocyst hatching failure. The limitation of our current study is the lack of more direct Nedd8 downstream regulation which will be deeply discovered in our further investigation. The low material amount in embryo samples makes it difficult to perform multiomics study at the protein or metabolite level, for example the conventional mass spectrometry. Considering the nature of neddylation as a post-translational modification, novel research strategies and state-of-the-art technologies would be employed.

## Materials and Methods

### Ethics Statement

All the animal protocols were approved by the Ethical Committee of Laboratory Animals and the Animal Care and Use Committee of Nanjing Medical University, and all the experiments were conducted following the Institutional Guide for the Care and Use of Laboratory Animals.

### Culture and Collection of Embryos

Female ICR mice (5-8 week) were super-ovulated by intraperitoneal injection of 5 IU of pregnant mare serum gonadotrophin (Ningbo Second Hormone Factory, Ningbo, China). After 46-48 h, the mice were injected with 5 IU of human chorionic gonadotrophin (Ningbo Second Hormone Factory, Ningbo, China). Ovulated metaphase II (MII) oocytes were collected 14-16 h later from the ampullae of oviducts and placed in HTF (human tubal fluid, Easycheck, Nanjing, China). Spermatozoa were collected from the epididymides of adult ICR male mice and pre-incubated in HTF for 1 h under mineral oil (Sigma, St. Louis, Missouri, USA) cover at 37°C with 5% of CO_2_. After insemination, the fertilized oocytes were washed and transferred into warmed KSOM media (Easycheck, Nanjing, China) under mineral oil cover at 37°C with 5% CO_2_. When the blastocyst stage was reached about 4 days later, we made gradient concentration (0μM, 0.5μM 1μM, 2μM, and 5μM) of MLN4924 (MCE, Monmouth, New Jersey, USA) into the KSOM media to treat the blastocysts for 24 h. Then blastocysts were collected for morphological observation, immunofluorescence, quantitative PCR, and low-input RNA sequencing.

### RNA Extraction and Quantitative PCR (qPCR)

RNA extraction from dissociated embryos (five embryos per sample) and cDNA amplification were performed according to the CellAmp Whole Transcriptome Amplification kit manual (TaKaRa, Kyoto, Japan).

Quantitative PCR was performed using ChamQ Universal SYBR qPCR Master Mix (Vazyme, Nanjing, China) with a QuantStudio 7 Real-Time PCR System (Applied Biosystems, Foster City, California, USA). Experiments were performed in triplicate. The qPCR primer sequences are shown in **Table 1**. Levels of target mRNAs were normalized to Gapdh and fold-induction of transcripts was calculated using the ddCT method.

**Table 1:** Quantitative PCR primer sequences.

Gene	Forward Sequence	Reverse Sequence
Bglap3	CCCTGAGTCTGACAAAGCCTTC	ACAAGCAGGGTCAAGCTCAC
Becn2	GACCCATCTGAACACGGAGG	CCTCTGGACTCTGGAAAACCTT
Cd68	ACTTCGGGCCATGTTTCTCT	GGGGCTGGTAGGTTGATTGT
Dppa1	GTGTCTGCTGCATTGCTGAT	CATGGAAGACAACCGAGCTTTC
Gsr	GCCAACAAAGAGGAAAAGGTGG	TGGCAACTGTGTTGTCGAAGT
Gclm	ACTCACAATGACCCGAAAGAAC	CCTGCTCTTCACGATGACCG
Nppb	AGTCCTTCGGTCTCAAGGCA	CCGATCCGGTCTATCTTGTGC
Prdx6	AAGCTGTCTATCCTCTACCCTG	GTGGGAACTACCATCACGCT
Txnrd1	TGCAACCTTAAAGACGATGAACG	TAGTCAGCCCACACTTGAGC
Gapdh	TGGCCTTCCGTGTTCCTAC	GAGTTGCTGTTGAAGTCGCA

### Immunofluorescence Staining

For immunofluorescence, embryos were fixed in 2% paraformaldehyde in PBS (pH 7.4) for 20 min and permeabilized using 0.5% triton X-100 in PBS for 20 min. The embryos were then incubated in blocking buffer (3% BSA in PBS) for 2 h followed by incubating with primary antibodies (diluted in blocking buffer) at 4°C overnight. After washing three times (15 min each) in washing buffer (0.5% tween-20, 0.5% triton X-100 in PBS), appropriate diluted fluorescent secondary antibodies were applied for 2 h. Embryos were then incubated in DAPI staining solution for 10 min. Apart from specially mentioned, all above procedures were performed at room temperature. The immunofluorescence staining was visualized by SP8 laser confocal microscope system (Leica, Wetzlar, Germany). Antibodies used are shown in **Table 2**. Nuclear was stained with DAPI staining solution (1:1,000, Beyotime, Shanghai, China, C1005). Quantification was performed by ImageJ.

**Table 2:** Immunofluorescence antibodies.

Antibody	Brand	Catalog Number	Ratio
IL-1β	Proteintech	16806-1-AP	1:400
IL-1ra	Abcam	ab124962	1:200
IL-6	Proteintech	66146-1-lg	1:100
p65	Proteintech	66535-1-Ig	1:400
IκB	CST	2859T	1:200
Nedd8	Abcam	ab81264	1:100
γ-H2AX	CST	9718T	1:400
Bcl2	Proteintech	26593-1-AP	1:400
BAX	Proteintech	60267-1-Ig	1:400
β-tubulin	Sigma	T5201-100ul	1:500
Alexa 488	Thermo	A11034	1:1000
Alexa 594	Thermo	A-11032	1:1000

### Low Input RNA Sequencing and Data Analysis

Blastocysts were collected 24 h after treatment with MLN4924 (five embryos per sample, three biological repeats in each group). The samples were stored in lysis buffer and transported with dry ice. Smart-seq2 was performed and sequenced by Annoroad, Nanjing. After the libraries passed quality checks, Illumina Novaseq PE150 was used for high-throughput sequencing. The quality control of sequencing data was assessed by FastQC (v0.11.9). The raw RNA-seq reads were mapped to the mouse genome (mm10) by STAR (v2.7.6a). Subsequently, they were quantified with featureCounts and the resulting quantization file was further evaluated with R (v4.0.2). Abnormal outlier sample (MLN3) was excluded by correlation analysis and principal component analysis (PCA). Then, DESeq2 package was used to identify differentially expressed genes (DEGs) and we considered genes with P adj < 0.05 and absolute value of log_2_FoldChange > 1 as DEGs. Gene ontology (GO) enrichment analysis and Kyoto Encyclopedia of Genes and Genomes (KEGG) pathway enrichment of DEGs were analyzed using the clusterProfiler R package. In addition, this package is also performed to gene set enrichment analysis (GSEA). The screening conditions were adjusted for P < 0.05 and q < 0.2. Web gene ontology annotation plot (WEGO) website (https://wego.genomics.cn/) was employed to compare GO annotation results and select the intriguing terms for further research. The protein-protein interaction (PPI) network was built by STRING database (https://string-db.org/), and CytoHubba plugin in Cytoscape (v3.9.0) was used to determine the hub genes.

### TUNEL Assay for Apoptosis Detection

Apoptosis in mouse blastocysts was detected using the TUNEL BrightGreen Apoptosis Detection Kit (Vazyme, China). Fresh blastocysts were fixed in 4% paraformaldehyde at room temperature for 25 min. After fixation, the embryos were washed in PBS and permeabilized by incubation in 0.5% Triton X-100 for 5 min at room temperature. The blastocysts were then washed two times in PBS and incubated with 50 μL TUNEL staining solution (1 μL Recombinant TdT enzyme, 10μL 5 × Equilibration Buffer, 35 μL H_2_O and 5 μL BrightGreen Labeling Mix) in the dark for 1 h at 37°C. After being counterstained with 5 μg/mL DAPI for 10 min at room temperature, blastocysts were washed in PBS, mounted with slight coverslip compression, and examined using SP8 laser confocal microscope (Leica, Germany). Blue fluorescence after DAPI staining showed the cell nuclei of the blastocyst, and green fluorescence indicated the cells that had undergone apoptosis.

### Measurement of Reactive Oxygen Species Generation

The generation capability for reactive oxygen species (ROS) was measured intracellularly using laser confocal microscope, which used the ROS probe 2′,7′-dichlorofluorescin diacetate (DCFH-DA) (Beyotime, China). Blastocysts were incubated with DCFH-DA (10 μmol/L) at 37°C for 20 min, which were then imaged by fluorescence microscope (excitation wavelength: 488 nm, emission wavelength: 525 nm) after washing again with MEMα medium.

### Adsorption of Trypan Blue Experiment

Blastocysts were incubated with Trypan blue (Biosharp, China) at final concentrations 0.4%(v/v) (in MEMα) for 3 minutes at room temperature (RT). Blastocysts were washed with MEMα again after Trypan blue treatment, which were then imaged by microscope.

### Statistical Analysis

All experiments were repeated at least three times to assure consistency. Means and standard deviations were plotted. Statistical analysis was performed using Student’s t-test and ANOVA. The significance level was P<0.05.

## Data Availability Statement

Sequence data that support the findings of this study have been deposited in BioProject with the primary accession code PRJNA778617.

## Ethics Statement

The animal study was reviewed and approved by Ethical Committee of Laboratory Animals and the Animal Care and Use Committee of Nanjing Medical University.

## Author Contributions

GY and JC contributed equally to this study. GY did the embryo-related experiments. JC analyzed data and drew relating figures. YH, HL, HY, and LC helped collecting fallopian tubes. LH, FM, and SH helped with molecular biology experiments. RF, CM, and YQ designed the study. RF, GY, and JC wrote and revised the manuscript. All authors reviewed and approved. All authors contributed to the article and approved the submitted version.

## Funding

This research was financially supported by grants from National Natural Science Foundation of China (81971451, 31900605), Nature Science Foundation of Jiangsu Province (BK20190654), Innovative and Entrepreneurial Team of Jiangsu Province (JSSCTD202144) and Innovative and Entrepreneurial Talent Program of Jiangsu Province.

## Conflict of Interest

The authors declare that the research was conducted in the absence of any commercial or financial relationships that could be construed as a potential conflict of interest.

## Publisher’s Note

All claims expressed in this article are solely those of the authors and do not necessarily represent those of their affiliated organizations, or those of the publisher, the editors and the reviewers. Any product that may be evaluated in this article, or claim that may be made by its manufacturer, is not guaranteed or endorsed by the publisher.
