# Supplementary figures and images for: Neddylation Inhibition Causes Impaired Mouse Embryo Quality and Blastocyst Hatching Failure Through Elevated Oxidative Stress and Reduced IL-1β
Source: Front Immunol. 2022 Jul 4;13:925702. doi: 10.3389/fimmu.2022.925702 (PMC9289163; doi:10.3389/fimmu.2022.925702)

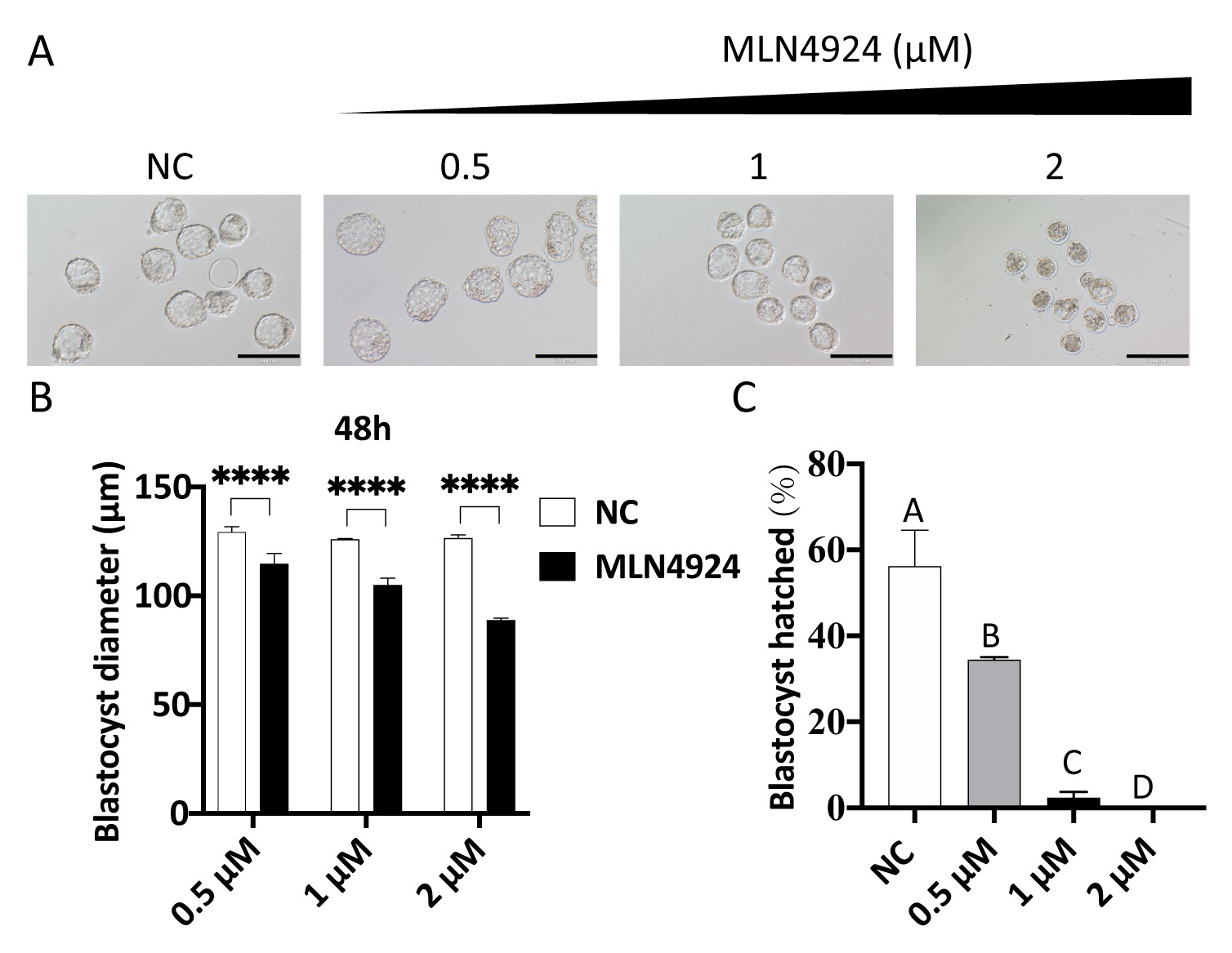

Supplement: Supplementary file 1 [file Image_1.tif]

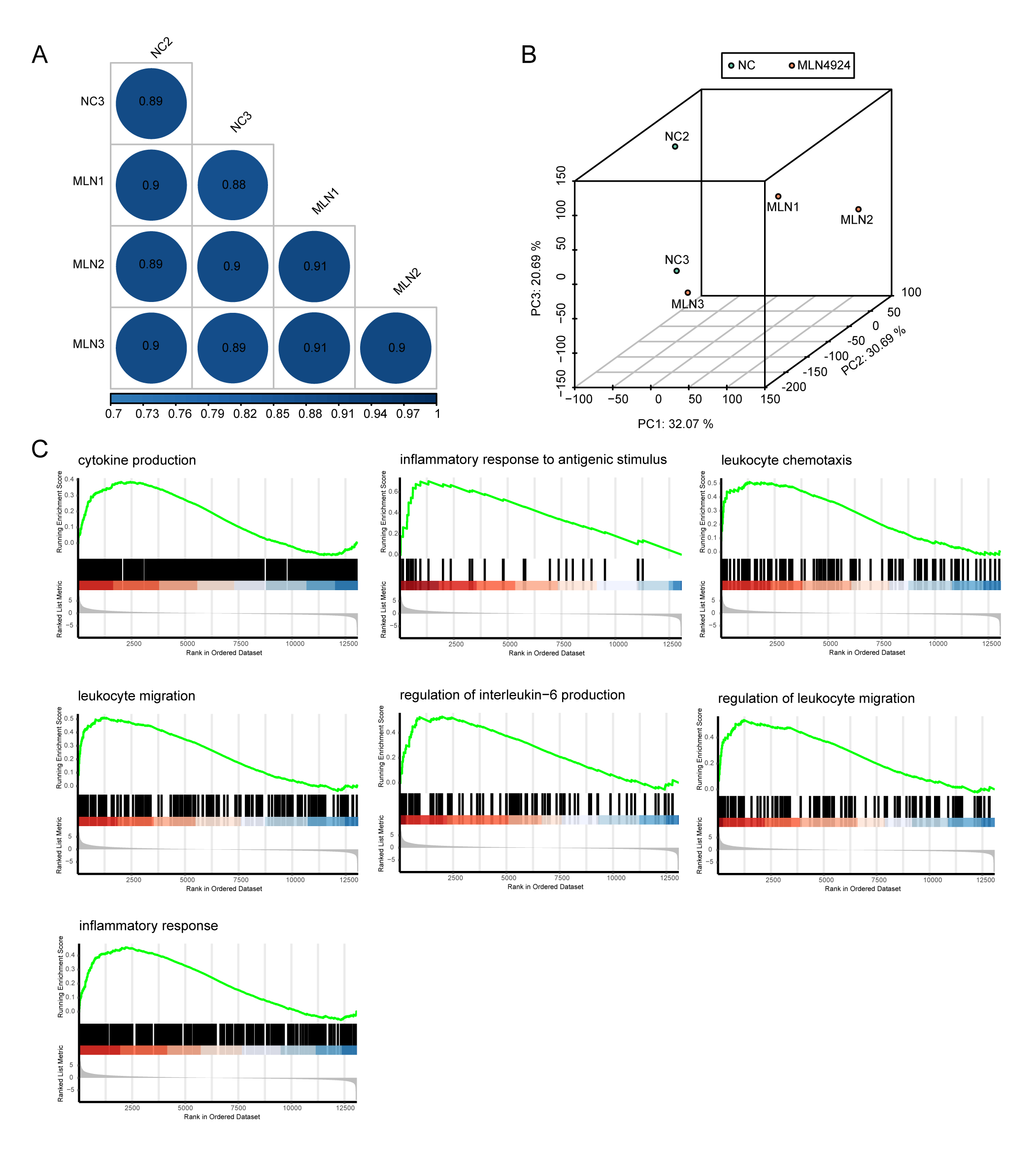

Supplement: Supplementary file 2 [file Image_2.tif]
